# Protective Role of Mast Cells in Primary Systemic Vasculitis: A Perspective

**DOI:** 10.3389/fimmu.2017.00990

**Published:** 2017-08-23

**Authors:** Jason M. Springer, Vineesh V. Raveendran, Selina A. Gierer, Mehrdad Maz, Kottarappat N. Dileepan

**Affiliations:** ^1^Division of Allergy, Clinical Immunology and Rheumatology, Department of Medicine, University of Kansas Medical Center, Kansas City, KS, United States

**Keywords:** mast cell, interleukin 6, suppressor of cytokine signaling-1, vasculitis, giant cell arteritis, aortitis, regulatory T-cell

## Abstract

Mast cells are important cells of the immune system. Although traditionally considered as key players in allergic and hypersensitivity reactions, emerging evidence suggests that mast cells have many complex roles in vascular disease. These include regulation of vasodilation, angiogenesis, activation of matrix metalloproteinases, apoptosis of smooth muscle cells, and activation of the renin angiotensin system. Mast cells are also known to play an immunomodulatory role *via* modulation of regulatory T-cell (Treg), macrophage and endothelial cell functions. This dual role of the mast cells is evident in myeloperoxidase anti-neutrophil cytoplasmic antibodies-mouse model of glomerulonephritis in which mast cell deficiency worsens glomerulonephritis, whereas inhibition of mast cell degranulation is effective in abrogating the development of glomerulonephritis. Our previous work demonstrated that mast cell degranulation inhibits lipopolysaccharide-induced interleukin 6 (IL-6) production in mice. This effect was not seen in histamine-1-receptor knockout (H1R^−/−^) mice suggesting a role for histamine in IL-6 homeostasis. In addition, mast cell degranulation-mediated decrease in IL-6 production was associated with an upregulation of suppressor of cytokine signaling-1 protein in the aorta. We propose that mast cells regulate large artery inflammation through T-cells, shifting a primarily Th1 and Th17 toward a Th2 response and leading to enhanced IL-10 production, activation Treg cells, and the inhibition of macrophage functions.

## Introduction

Vasculitis refers to inflammation of blood vessels, typically of the arterial circulation. The pathogenesis of vasculitis varies by type, but typically involves recruitment and activation of T-cells and macrophages with resultant generation of a variety of inflammatory cytokines including IL-6. Primary systemic vasculitis is classified based on the size of the blood vessels predominantly affected (1). Primary large vessel vasculitis can affect the aorta and any of its proximal branches. Giant cell arteritis (GCA), a large vessel vasculitis, is the most common form of primary systemic vasculitis, with an annual incidence of 25 per 100,000 in those over the age of 50 (2). Takayasu’s arteritis (TAK), another form of large vessel vasculitis, usually occurs before age 50. Because of the striking similarity in the pattern of arterial involvement, speculation has emerged regarding whether GCA and TAK represent a spectrum of the same disease (3). Polyarteritis nodosa (PAN) is a necrotizing arteritis of medium to small vessels. Most commonly, PAN affects medium sized arteries of the skin, kidneys, peripheral nerves, and the gastrointestinal tract. Kawasaki’s disease, another form of medium to small vessel vasculitis which almost exclusively occurs in pediatric populations, is associated with mucocutaneous lymph node syndrome, and frequently affects the coronary arteries leading to coronary artery aneurysms. Small vessel vasculitis is classified into different phenotypes such as immune-complex mediated disease (i.e., cryoglobulinemic vasculitis, IgA vasculitis, and hypocomplementemic urticarial vasculitis) and anti-neutrophilic cytoplasmic antibody (ANCA)-associated forms [i.e., granulomatosis with polyangiitis (GPA), microscopic polyangiitis (MPA), eosinophilic granulomatosis with polyangiitis (EGPA), and renal limited vasculitis].

## Mast Cell Physiology

Mast cells are primarily known for their functions in allergy, hypersensitivity reactions, asthma-associated vasodilation, bronchoconstriction, and angioedema. Based on the microenvironment in which mast cells reside they are classified into two distinct phenotypes namely, mucosal type (M-MC) and connective tissue type (CT-MC). M-MC and CT-MC are also known as tryptase-positive and chymase/tryptase-positive mast cells, respectively. Other distinct characteristics of these mast cell subsets are that M-MC granules contain lesser amounts of histamine and use chondroitin sulfate as the core proteoglycan, whereas CT-MC granules store 10–15 times higher histamine and contain heparin as the packaging matrix (4). It should be emphasized that these MC phenotypes are better characterized in rodents than in humans, although the heterogeneity is evident in both species.

Classic mast cell degranulation is triggered by the interaction of antigen-specific IgE with FcεRI receptors located on mast cells. However, mast cells can also secrete many inflammatory and immunomodulatory mediators without degranulation. These events are initiated by a host of other stimuli including IgA, IgG, adenosine, C3a, chemokines, cytokines, pathogen-associated molecular patterns (PAMPs), and toll-like receptor (TLR) ligands. The response of the mast cell to these stimuli can vary.

For instance, TLR2 activation through PAMPs derived from Gram-positive bacteria can result in mast cell release of IL-4. Whereas TLR4 activation through Gram-negative bacterial agents does not result in mast cell degranulation, but causes the release of proinflammatory cytokines including tumor necrosis factor alpha (TNFα), IL-1, and IL-6 (5).

**Figure 1:**
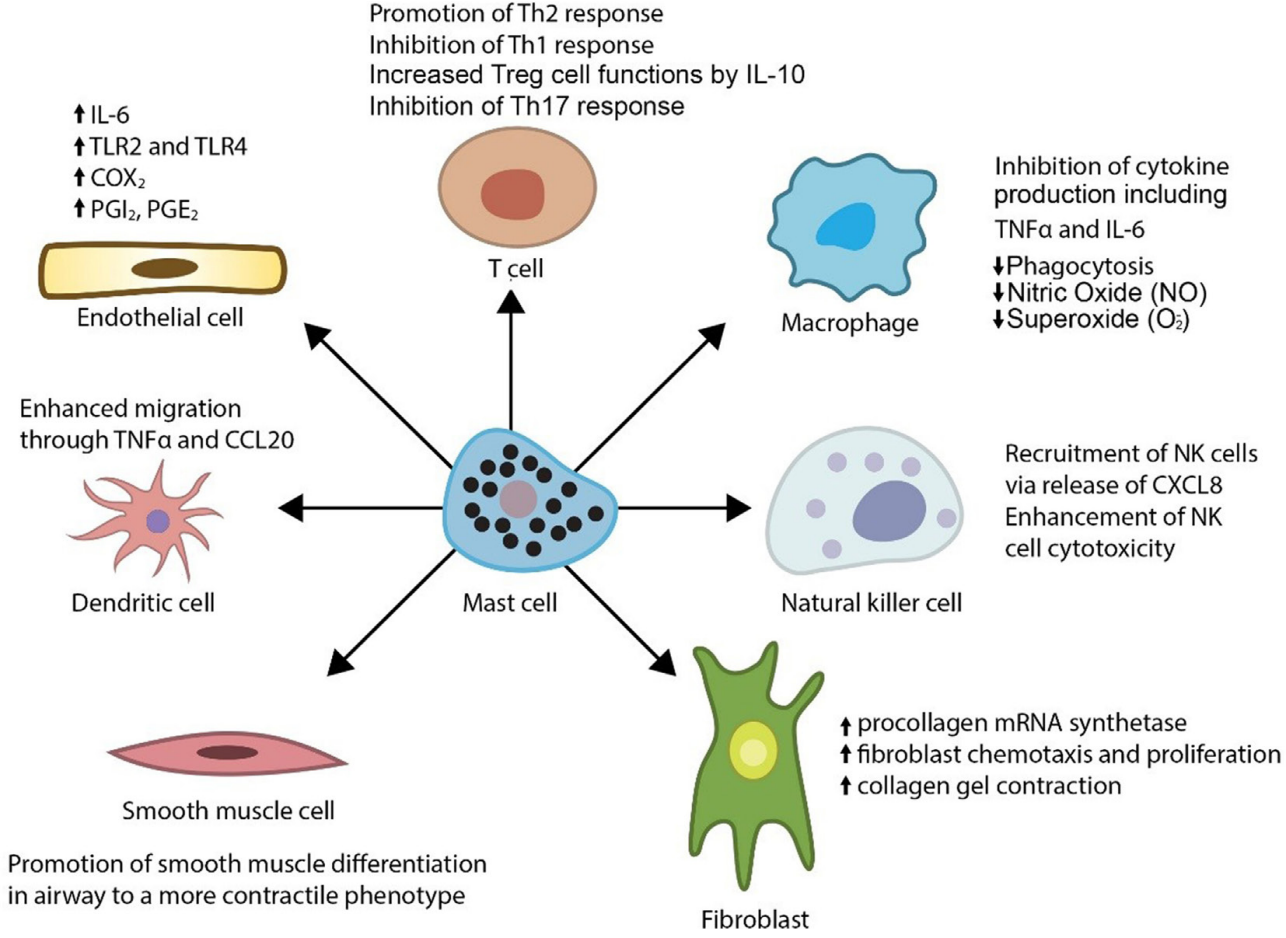
Effects of mast cells on immune and vascular cells.

**Figure 2:**
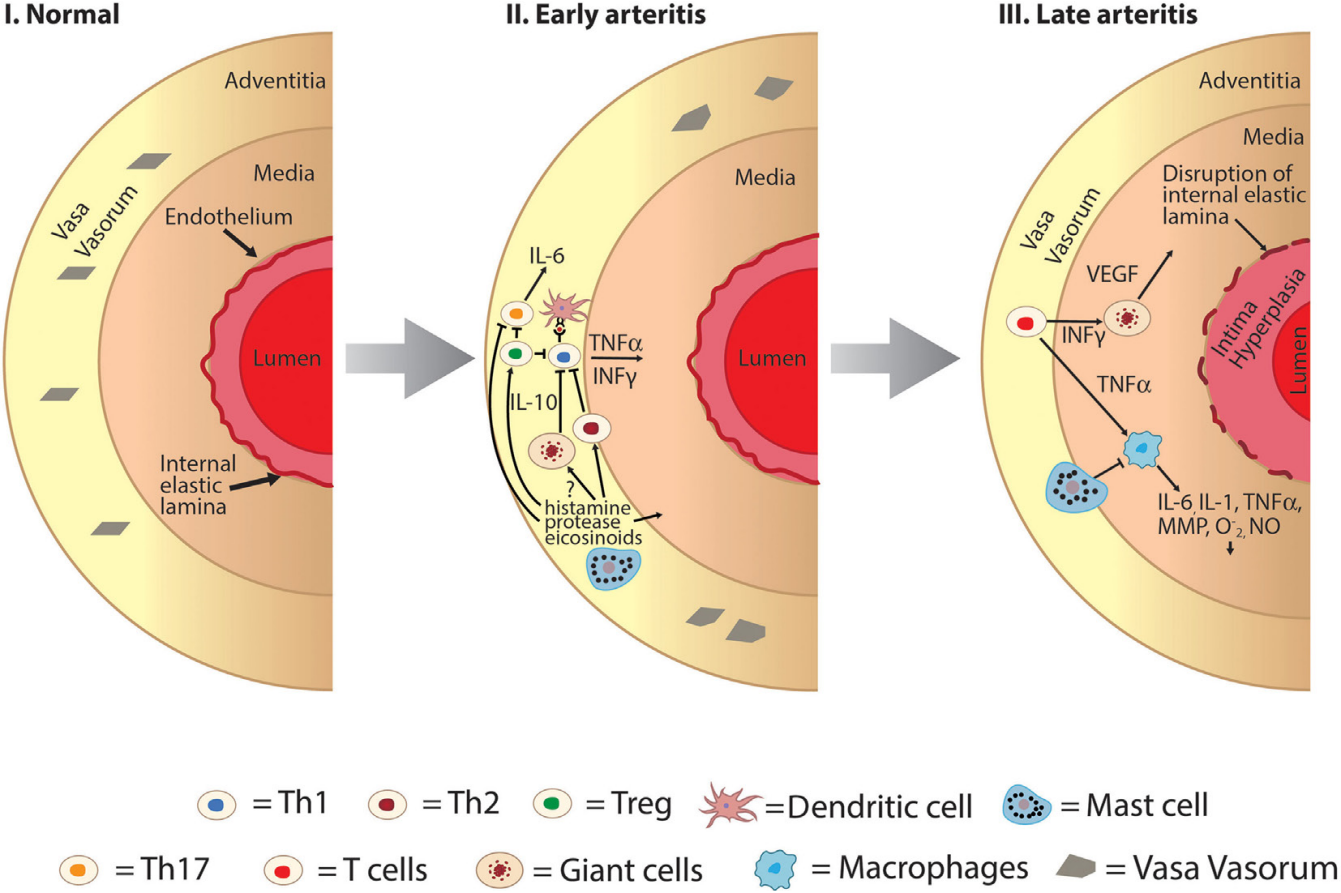
Hypothesized role of mast cell as an immunomodulator in the development of large vessel vasculitis. I: normal large vessel artery. II: mast cells may play a protective role in early arteritis through inhibitory effects on Th1 and Th17 pathways and activation of Th2 pathways. Mast cell degranulation results in promotion of regulatory T-cell cells which also inhibits both Th1 and Th17 cells. III: later in the pathogenesis of large vessel vasculitis structural changes of the vessel wall occur including intimal hyperplasia, medial thickening, and disruption of the internal elastic lamina. Mast cells may mediate late arteritis through inhibition of macrophage-mediated propagation of large vessel vasculitis including decreased production of key cytokines such as TNFα and IL-6, decreased phagocytosis, and production of superoxide and nitric oxide (${\text{O}}_{2}^{-}$, NO). INFγ, interferon gamma; IL, interleukin; MMP, matrix metalloproteinases; NO, nitric oxide; ${\text{O}}_{2}^{-}$, superoxide; Th, T-helper cells; Treg, T-regulatory cells; TNFα, tissue necrosis factor alpha; VEGF, vascular endothelial growth factor.

In addition, mast cells release a myriad of other vasoactive and inflammatory substances including histamine, proteases (tryptase, chymase, and carboxypeptidase A), prostaglandin D2, leukotrienes, heparin, and various cytokines including IL-10. Physiologically, mast cells are known regulators of a variety of vascular cell types (Figure 1) (6–10) and cause vasodilation, angiogenesis, and elimination of foreign biotics including bacteria and parasites. Thus, the mast cell has been considered as a pluripotent “master cell,” with a predominant role in vascular inflammation and vascular homeostasis (10). In this article, we present our perspective on the mast cell involvement in systemic vasculitis and propose a novel inhibitory mechanism through which mast cells control large artery inflammation (Figure 2).

## Mast Cells and the Artery

Mast cells are strategically located in either mucosal surfaces or connective tissues. Histologically, mast cells reside in close proximity to arterial vessels. Mast cells are found in the media and adventitia of the aorta. In experimental animal models of abdominal aortic aneurysms (AAAs), mast cell-deficient mice are less prone to developing AAAs (11, 12). There are several functions of mast cells that likely account for aneurysmal development including activation of matrix metalloproteases, apoptosis of smooth muscle cells through granzyme B (13), and activation of the renin angiotensin system.

Mast cells are found near the vasa vasorum in the adventitia of coronary arteries of patients with atherosclerosis and play a role in plaque stability (14). Systemic activation of mast cells results in increased progression of atherosclerotic plaque formation in apoE-deficient mice and mast cell stabilization with cromolyn can prevent plaque rupture (15). Proteases released from the mast cell can degrade collagen in the atheroma, which can predispose the plaque to rupture (16). Subsequently, endogenous protease inhibitors suppress the protease activity and promote apoptosis of macrophages, which leads to increased necrotic core size of plaques compromising their instability (15).

Spontaneous coronary artery dissection (SCAD) is a rare cause of acute myocardial ischemia or sudden cardiac death, accounting for an estimated 0.1–0.28% of cases (17). Eosinophilic coronary periarteritis (ECPA) has a strong association with SCAD and is reported to account for up to 40% of cases. Examination of dissected coronary arteries from ECPA cases reveals a threefold higher adventitial mast cell density in dissected portions of the artery as well as a trend toward increased degranulation (18). Mast cell-derived arterial vasospastic mediators are thought to contribute to the SCAD in ECPA cases (19).

## Small Vessel Vasculitis

Mast cells play a complex role in vascular homeostasis since they synthesize, store, and secrete both pro- and anti-inflammatory molecules. This phenomenon is best demonstrated in human and animal studies of ANCA-associated vasculitis. Human disease can phenotypically be divided into subsets. MPA presents with features of capillaritis without granulomatous formation including, most commonly, diffuse alveolar hemorrhage, and pauci-immune glomerulonephritis. In addition to features of capillaritis, GPA presents with features of granuloma formation including lung nodules and retro-orbital disease. EGPA presents with features of capillaritis and granuloma formation along with asthma and eosinophilia. The pathogenesis of ANCA-associated vasculitis is thought to start with priming of neutrophils and monocytes by inflammatory stimuli. This in turn leads to the display of antigens, such as myeloperoxidase (MPO) and proteinase 3, on the cell surface which can then interact with ANCAs causing neutrophil activation, vascular inflammation, and granuloma formation (20). Neutrophil extracellular traps (NETs) are extracellular web-like DNA structures containing antimicrobial proteins such as MPO, which have recently been implicated in the pathogenesis of ANCA-associated vasculitis. NET formation has been observed in kidney biopsies from patients with ANCA associated vasculitis (21). Inhibition of peptidylarginine deiminase 4, important in citrullination of histones yielding decondensation of DNA and suicidal NETosis, results in inhibition of MPO-ANCA formation *in vivo* (22).

A mouse model mimicking glomerulonephritis seen in human ANCA-associated vasculitis has been developed in which mice are immunized with MPO followed by passive transfer of low dose anti-glomerular basement membrane antibodies, which then induces focal segmental glomerulonephritis. Gan et al., utilizing this model, have shown that mast cell deficient mice (Kit^W-sh/W-sh^) exhibit more anti-MPO CD4^+^ T cells, a stronger delayed hypersensitivity response to MPO, and more severe glomerulonephritis compared with wild-type mice (23). Furthermore, mast cell-deficient mice express fewer regulatory T-cell (Tregs) and reduced production of IL-10 from lymph nodes. Reconstitution of mast cells from wild-type control mice, but not from IL-10 deficient mast cells, significantly increased the Treg numbers and attenuated the severity of the glomerulonephritis (23). Subsequent work by the same group of investigators using human kidney biopsies from patients with positive MPO-ANCA and focal segmental glomerulonephritis demonstrated a higher density of interstitial mast cells (degranulated and spindle shaped mast cells) compared with disease controls (thin basement membrane disease or adult minimal change disease) (24). Mast cells have been found to be prominent producers of IL-17 in the human kidney based on evaluation of biopsies (25). Administration of disodium cromoglycate (DSCG), a mast cell stabilizing agent that acts by targeting calcium channels and inhibiting mast cell degranulation (26), attenuated T-cell responses with associated decreases in interferon gamma (IFNγ) and IL-17A production while promoting IL-10 production. Furthermore, DSCG inhibited the development of glomerulonephritis and mast cell presence within the kidneys. DSCG administration to mast cell deficient mice had no effect on IFNγ, IL-17A, or the development of glomerulonephritis providing evidence that these effects are driven through DSCG-induced stabilization of mast cells (24).

Whether mast cells stimulate or inhibit the immune system is dependent on the physiological milieu of the mast cells and the microenvironment in which they reside. Mast cells release preformed mediators immediately upon immune (e.g., antigen-IgE complexes, complement, and immune aggregates) and non-immune (e.g., radiocontrast, medication, exercise, and cold air) induced degranulation. Mast cells can also be activated through TLR4 to synthesize and release a variety of pro-inflammatory mediators including TNFα, IL-6, IL-1, and IL-13 (5). Interestingly, various stimuli have been reported to prevent mast cell degranulation or generate anti-inflammatory mediators. For instance, stimulation of vitamin D receptors results in the release of IL-10 by mast cells without causing degranulation (27). Similarly, DSCG appears to promote mast cell production of IL-10 while inhibiting degranulation. In the ANCA-associated mouse model of glomerulonephritis, DSCG was able to attenuate Th1 and Th17 pathways, which play a dominant role in the pathogenesis of ANCA-associated vasculitis. It is unclear if this is primarily mediated through inhibition of mast cell degranulation or through the production of IL-10. The direct effects of mast cells on other innate immune cells, such as neutrophils, in ANCA-associated vasculitis are not clearly defined. In addition, like neutrophils, mast cells can produce antimicrobial extracellular traps (MCET) (28). The putative effects of MCET on the pathogenesis of small vessel vasculitis are yet to be explored.

## Large Vessel Vasculitis

Very early on during the pathogenesis of large vessel vasculitis, inflammatory cells enter the vessel wall through the vasa vasorum of the arterial adventitial layer. Activation of TLR4 and TLR5 on dendritic cells leads to the orchestration of the inflammatory cascade within the vessel wall (29). Dendritic cells recruit T-cells and macrophages using a variety of chemokines (CCL 18, 19, 20, and 21); secrete important cytokines to sustain inflammation including IL-1β and IL-6; and promote pro-angiogenic factors such as vascular endothelial growth factor, platelet-derived growth factor, and fibroblast growth factor. Both Th1 and Th17 cells contribute to the pathogenesis of GCA. Th1 cells release IL-12 and IFN-γ, whereas Th17 cells release IL-17 upon differentiation when stimulated by TGF-β, IL-6, IL-21, and IL-23. Ultimately, these early events will result in structural changes of the arterial wall including disruption of the internal elastic laminal and endothelial proliferation. While glucocorticoids significantly affect the Th17 cytokine signature, IFN-γ is relatively unaffected (30). Randomized controlled trials for methotrexate and TNFα inhibitors in the treatment of GCA have all been disappointing (31–33). Furthermore, autopsy studies have demonstrated clinically unrecognized smoldering large vessel vasculitis (34). It is noteworthy that, prior to the widespread use of glucocorticoids for the treatment of GCA, a case report documented the beneficial effect of intravenous histamine in one patient (35).

Mounting clinical evidence supports the role of IL-6 as a dominant cytokine in the pathogenesis of GCA. Serum IL-6 levels are significantly higher in patients with GCA and more sensitive than traditional biomarkers in determining disease activity in treated and untreated patients (36). Tocilizumab is a humanized antibody which targets the IL-6 receptor and blocks IL-6 signaling. In a small cohort of nine refractory/relapsing patients with large vessel vasculitis treatment with tocilizimab lead to clinical and serological improvement as well as a significant reduction in the average dose of prednisone in seven patients with GCA (37). One patient had persistent large vessel vasculitis at autopsy despite the appearance of clinical remission, which stresses the difficulty of assessment of active disease in this patient population. Randomized controlled trials since that time have further supported IL-6 inhibition for the treatment of GCA (38).

Our published work has demonstrated a novel phenomenon that mast cell-derived histamine and lipopolysaccharide (LPS) (a TLR4 ligand) synergistically enhance IL-6 gene expression and cytokine production in human umbilical vein endothelial cells and in coronary artery endothelial cells *in vitro* (39–42). These findings highlight an important role for mast cells in IL-6 homeostasis in the vasculature. Inhibition of histamine receptor -1 (H1R), but not H2R signaling, was found to abrogate this effect indicating that histamine-induced enhancement of IL-6 production by endothelial cells is mediated through H1R stimulation (42–44). In addition, LPS was found to induce the expression of H1R and histamine stimulated the expression of TLR4 in endothelial cells (42, 44). These findings suggest that histamine and bacterial agents act in concert to amplify endothelial inflammatory responses in a bi-directional way. While the *in vitro* evidence shows mast cell-induced enhancement of arterial wall inflammation, clinical and *in vivo* studies suggest a much more intricate and dynamic role. For instance, C57Bl/6J mice injected with the combination of LPS and a mast cell degranulation agent showed reduced serum IL-6 levels, as well as diminished aortic expression of IL-6 mRNA when compared with mice injected with LPS alone after 24 h. This IL-6 lowering phenomenon mediated by mast cell degranulation was associated with the upregulation of aortic expression of suppressor of cytokine signaling (SOCS)-1 (45). SOCS proteins act as negative inhibitors of intracellular inflammatory pathways by reducing the expression of many pro-inflammatory cytokines. Of special emphasis is that, SOCS-1 has a role in inhibiting IL-6 targeted inflammatory pathways through JAK-STAT signaling inhibition (46). However, these effects are likely acute as wild-type mice injected serially over a 10-day period had no significant increase in IL-6 production when receiving LPS injections and the inhibitory effects of mast cells were not evident. In contrast, over the 10-day time period, there were significantly higher levels of IL-10 suggesting that other regulators of inflammation play a more dominant role, such as Treg cells. The inhibitory effect of mast cell degranulation on aortic expression of IL-6 was not seen in H1R knockout mice supporting that histamine/H1R axis may play a major role in this effect (unpublished data).

## Mast Cells as Regulators of Inflammation

Although mast cells secrete many pro-inflammatory agents including histamine, proteases, prostanoids, leukotrienes, and cytokine, they also release many anti-inflammatory agents that inhibit macrophage and T-cell functions. Our previous work has shown that mast cell granules downregulate LPS-induced production of superoxide, nitric oxide, and TNFα in macrophages *via* inhibition of NF-κB translocation/degradation (47). Our studies have documented that mast cell granules are phagocytosed by macrophages into phagolysosomes and rapidly degraded. This may lead to the release of its proteases into the cytoplasm (48) leading to or promoting consequent degradation of NF-κB proteins (49), which might explain the mast cell granule-mediated inhibition of LPS-induced TNFα and nitric oxide synthase (47). In contrast, mast cell granules as well as mast-cell-derived histamine and proteases synergistically enhanced LPS or TNFα-induced IL-6 production by endothelial cells (39–42). Although, further studies are warranted to define the precise mechanisms involved in the modulation of vascular inflammation by mast cell mediators, we speculate that the phenomenon is cell-specific and the regulation of NF-κB translocation might be a major contributing factor. The dual role of mast cells may serve key physiological functions such as maintenance of tissue homeostasis after a microbial or parasite infestation, prolonged inflammatory response, or extensive tissue injury. Our preliminary results demonstrating inhibition of LPS-induced aortic IL-6 gene expression and systemic IL-6 production in mice by acute mast cell degranulation support the notion that the mast cell may act as a potent negative modulator of vasculitis.

Increasing evidence also supports a role for mast cells in the regulation of T-cell responses. Mast cells can act as tissue localized antigen presenting cells to CD4^+^ T cells as demonstrated in inflamed human psoriatic skin (50). Stimulation of mast cells *in vitro* with LPS and IFNγ promotes the expression of MHC class II and co-stimulators (51). Important in the crosstalk of mast cells and CD4^+^ T cells is the costimulatory molecule OX40L on the surface of mast cells. Upregulation of the mast cell expression of OX40L has been reported to be due to exposure to TLR agonists, FcεR1 engagement, and soluble TNF (51, 52). Thus, the interaction between T-cell-associated OX40 and mast cell-associated OX40L contribute to T-cell proliferation and cytokine secretion. The crosstalks between mast cells and CD4^+^ T cells result in differentiation into Th2 cells producing IL-4, IL-5, IL-10, and IL-13. Furthermore, mast cells expressing MHC class II have been shown to preferentially expand antigen-specific T cells over naïve T cells (51). Mayranpaa and colleagues evaluated human temporal artery specimens obtained from patients with GCA for the presence of mast cells and T cells. Mast cells immunostaining positive for active tryptase, cathepsin G, and vascular endothelial cell factor were found to be associated with increased T cells. In addition, mast cells accumulated around neointimal neovessels in the GCA lesions (53).

## Conclusion and Perspective

Mast cells are important players in vascular homeostasis. In systemic forms of vasculitis mast cells play a role in both pro-inflammatory and immune-regulatory responses. Emerging evidence supports that mast cell responses can be mediated through effects by the surrounding milieu and inflammatory cells. An example of this, seen by our group, is the pro-inflammatory effects of mast cell mediators (e.g., histamine and proteases) on isolated endothelial cells *in vitro*, but inhibitory effects on the aorta *in vivo* (as measured by IL-6). We suspect that these inhibitory effects are driven by the interaction of mast cells with other inflammatory cells such as T-cells and macrophages. In addition, the overall inflammatory effects may vary dramatically based on whether mast cell degranulation has occurred. In the MPO-ANCA vasculitis mouse model, mast cell deficiency worsens ANCA mediated glomerulonephritis, whereas inhibition of mast cell degranulation with DSCG inhibits the development of glomerulonephritis. Indeed, certain stimuli are known to promote mast cell release of selective cytokines, such as IL-10, without causing degranulation. Thus, it is possible that mast cell products derived from active degranulation and spontaneous secretion may exert distinctively different modulatory effects.

In this perspective article, we propose a mechanism by which mast cells may inhibit progression of large vessel vasculitis. In our model, mast cell degranulation inhibits aortic expression of IL-6 mRNA and decreases serum levels of IL-6, an important cytokine in the pathogenesis of large vessel vasculitis. Although mast cells spontaneously and upon degranulation release a wide array of pro- and anti-inflammatory mediators, histamine is considered as a major player that influences vascular tone in many ways. The role of histamine in inhibiting aortic IL-6 production is supported by the loss of this regulatory effect in H1R knockout mice (preliminary data). H1R is found on numerous cells including T-cells, and histamine is known to drive naïve T-cell differentiation toward a primarily Th2 response. This, in turn, will inhibit Th1 responses, which are known to be a prominent inflammatory pathway in large vessel vasculitis. Proteases, released during degranulation, have a similar effect on naïve T-cells. Intracellularly, we suspect that stimulation of the H1R causes upregulation of SOCS-1, which inhibits Th1 responses through effects on JAK-STAT pathways, although this remains to be tested. Macrophages also play an important role in the pathogenesis of large vessel vasculitis. Since mast granules and histamine are known to inhibit macrophage functions *in vitro* (49, 54, 55), we speculate that mast cell degranulation also limits macrophage production of IL-6. Collectively, we propose that mast cell mediators, especially histamine, may play a major role in the inflammatory homeostasis in primary systemic vasculitis, including large vessel vasculitis.

## Ethics Statement

All our work cited here were approved by the Institutional Animal Care and Use Committee (IACUC) at the University of Kansas Medical Center.

## Author Contributions

JS and KD developed the concepts and prepared the initial draft of the manuscript. VR, JS, and KD developed the experimental designs, generated data, and analyzed the results. SG and MM provided consultation for special topics within the manuscript. All authors critically edited and reviewed the final manuscript.

## Conflict of Interest Statement

The authors declare that the research was conducted in the absence of any commercial or financial relationships that could be construed as a potential conflict of interest.
